# Cellular immunophenotyping in human and primate tissues during healthy conditions and Ebola and Nipah infections

**DOI:** 10.1172/jci.insight.185861

**Published:** 2025-04-17

**Authors:** Andrew P. Platt, Bobbi Barr, Anthony Marketon, Rebecca Bernbaum, Deja F.P. Rivera, Vincent J. Munster, Daniel S. Chertow, Michael R. Holbrook, Scott M. Anthony, Bapi Pahar

**Affiliations:** 1 Laboratory of Virology, National Institute of Allergy and Infectious Diseases,; 2 Emerging Pathogens Section, Critical Care Medicine Department, Clinical Center, and; 3 Critical Care Medicine Branch, National Heart, Lung, and Blood Institute, NIH, Bethesda, Maryland, USA.; 4 Integrated Research Facility at Fort Detrick, National Institute of Allergy and Infectious Diseases, NIH, Fort Detrick, Frederick, Maryland, USA.; 5 Laboratory of Virology, National Institute of Allergy and Infectious Diseases, NIH, Hamilton, Montana, USA.

**Keywords:** Immunology, Infectious disease, Cell migration/adhesion, T cells

## Abstract

We developed a 29-color spectral cytometry panel to enhance nonhuman primate (NHP) models for cross-reactive immunophenotyping. This panel is suitable for biosafety level 4 (BSL-4) viruses and can be used with both human and NHP samples in BSL-2 research settings. Tissues from humans, rhesus monkeys (RhMs), crab-eating macaques (CEMs), and green monkeys (GMs) were stained with a 29-color immunophenotyping panel requiring only 2 clone substitutions. Comparable staining was observed for all samples. Unbiased analysis showed acceptable overlap in T cell phenotypes across samples, with differences in human and NHP B cells and granulocytes. In CEMs, most circulating CD8^+^ T cells were from effector memory cells, with significantly higher levels than in humans, RhMs, and GMs. Analysis of samples from various anatomical sites revealed distinct location-specific phenotypes. In Nipah virus–exposed animals, splenocytes showed a substantial increase in IgM^+^ B cells and a reduction in effector memory CD8^+^ T cells compared with unexposed controls. Lymph nodes from Ebola virus–exposed animals showed a loss of CXCR3^+^CD8^+^ T cells versus unexposed controls. This panel may guide the development of additional multicolor panels in preclinical and clinical settings and may increase understanding of the pathogenesis of diseases caused by emerging and reemerging viruses.

## Abstract 2

Development of a flow cytometry panel that can be used for human and another non-human primate model to characterize different cell populations in healthy and highly infectious pathogenic viral diseases.

## Introduction

In recent decades, the world has experienced outbreaks caused by new and reemerging viruses, including SARS-CoV-2, monkeypox virus (MPXV), Ebola virus (EBOV), and Nipah virus (NiV), causing morbidity, excess mortality, and economic burden. Since December 2019, SARS-CoV-2 has caused over 777 million affected individuals and over 7 million deaths worldwide (1). Various clades of MPXV have caused widespread disease in central Africa, Europe, and the United States. The 2014–2016 EBOV disease (EVD) outbreak in Western Africa resulted in an estimated 28,600 affected individuals and 11,325 deaths. A September 2022 outbreak of disease caused by Sudan virus in Uganda resulted in 164 affected individuals and 55 deaths (2). A recent NiV infection outbreak in India resulted in 6 affected individuals and 2 deaths (3). NiV infection outbreaks in Bangladesh, India, Malaysia, and Singapore have had fatality rates of 40%–100. Given that many of these infections occur in remote settings with unpredictable timing, medical countermeasures need to be available. Relevant preclinical animal models are key to evaluating disease pathogenesis and testing vaccines and therapeutics. Understanding host immune responses to infection and correlates of protection in blood and tissues is essential to guide countermeasure development. High-dimensional flow cytometry is an invaluable tool for elucidating human and animal cellular immune responses during infection (4–7).

Nonhuman primates (NHPs) possess many biological, immunological, and genetic similarities to humans, making them valuable for investigating immune responses during the disease course. Rhesus monkeys (RhMs) are commonly used to study EBOV pathogenesis and therapeutics development (8, 9). Crab-eating macaques (CEMs) are useful for evaluating Sudan virus pathogenesis (10) as well as EBOV and NiV vaccine safety and efficacy (11, 12). Green monkeys (GMs) are among the best-suited animal models for studying the pathogenesis of NiV infection, evaluating vaccine candidates, and testing therapeutic efficacy (8). Spectral flow cytometry allows for deep characterization of integral cell populations in various tissues in human samples and animal models through panel complexity and use of autofluorescence. With a comprehensive flow cytometry panel, it is possible to observe essential immune cell interaction parallels of healthy controls and models of disease.

The creation of a comprehensive flow cytometry panel has had challenges, including obtaining cross-reactive antibodies for the relevant models and avoiding spectral overlap between fluorophores. In recent years, more cross-reactive antibodies have become available, and there have been significant advancements in instrumentation capabilities and the availability of fluorochromes to enable high-dimensional analysis. Despite these forward strides, accessing antibodies for advanced animal models, such as various NHP species, continues to have limitations (13). Furthermore, a lack of fluorochrome-labeled antibodies that intersect spectrally poses challenges, including spectral overlap, when optimizing a multifaceted flow cytometry panel. To bypass issues associated with animal model antibodies, the use of multidimensional flow cytometry panels with PBMCs has been on the rise. For example, a 28-color panel assesses the activities of different B cells and myeloid cells (14). Recently, high-parameter flow cytometry panels (including those for 40 and 43 colors; refs. 15, 16) were developed for deep immunophenotyping of human PBMCs. Nevertheless, all of those panels were created to target cell phenotypes in human samples and have not been tailored to NHP samples.

Cell immunophenotyping presents a challenge in comparing animal disease models to observations made in patients when using different clones of antibodies to define a cell population. Thus, we developed a 29-color flow cytometry panel that can analyze the immune cell composition of PBMCs and tissue samples from patients in the clinic and NHP animal models. This panel has been designed for research involving pathogens that require biosafety level 4 (BSL-4) containment (including EBOV and NiV). Our panel includes cross-reactive antibodies that work across human, RhM, CEM, and GM fresh and cryopreserved PBMCs, splenocytes, liver cells, and mesenteric lymph node (Mes LN) cells. Our panel of markers enables the evaluation of vital immune cells in various species without the need to adjust fluorophores. Only 2 antibody switches are required to analyze human cells. The markers in our panel enable precise identification of diverse subsets of lymphocytes, monocytes, macrophages, and DCs. This panel facilitates the evaluation of T, B, and NK cells, including their activation, proliferation, and differentiation stages. These cellular populations are crucial for establishing healthy baseline measurements and assessing disease progression in numerous high-consequence viral pathogens, as well as elucidating their role in vaccine-mediated protection. With its broad reactivity and optimized spectral overlap, our panel can easily accommodate additional markers of interest for specific studies, making it a suitable phenotyping panel with great potential to identify a broad range of cell populations.

## Results

### Differences in staining intensity among species.

A flow cytometry panel capable of distinguishing among various cell types (such as T cells, B cells, monocytes, DCs, and tissue-specific macrophages) was created (Table 1). This new panel consists of 29 colors and has an overall complexity index of 9.52. Four pairs of fluorochromes (BV421 and V450, APC and BUV661, BV510 and BV480, and BV786 and BV750) have similarity index matrices (SIMs) of 0.81, 0.82, 0.85, and 0.85, respectively (Table 1 and Supplemental Figure 1; supplemental material available online with this article; https://doi.org/10.1172/jci.insight.185861DS1), which are higher than the SIMs of all other fluorochromes. The panel was examined for compatibility with cells from different species and tissues infected with NiV or EBOV. To evaluate the species cross-reactivity of the panel, an initial comparison was made among PBMC samples from 4 species (human, *n* = 4; RhM, *n* = 3; CEM, *n* = 3; GM, *n* = 4) after gating down to live CD45^+^ singlet cells (Table 2 and Supplemental Figure 2). Individual histogram plots of each marker (Supplemental Figure 3) show the differences in staining intensity of some markers among species.

### Metacluster frequency difference among species.

**Figure 1:**
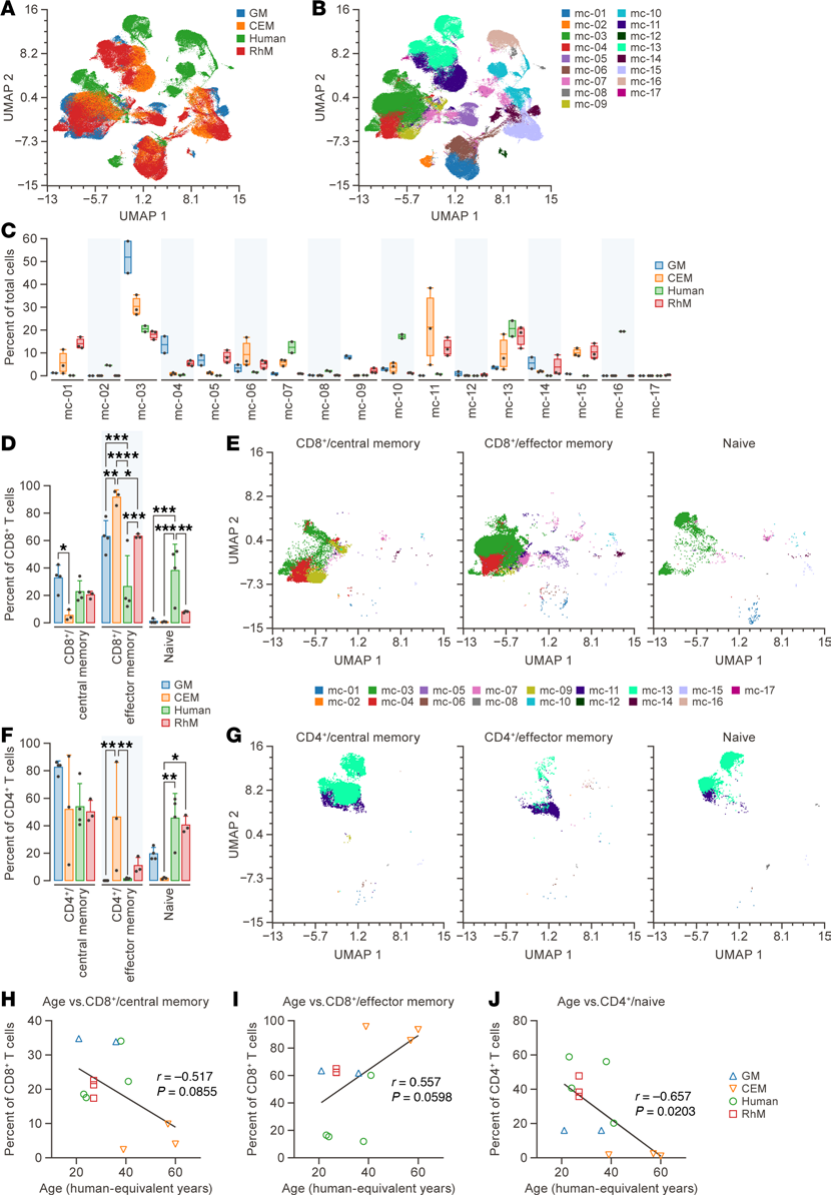
Cross-species comparisons. (**A**) UMAP dimensional reduction of PBMCs from human and 3 NHP species demonstrating overlap of major populations. Species identity is color coded. (**B**) FlowSOM metaclusters (*k* = 17) projected onto UMAP plot from **A**. (**C**) Proportion of total live CD45^+^ PBMCs for each species represented by each metacluster from **B**. (**D**) Proportion of CD8^+^ T cells made comprised of central memory (CD28^+^CD95^+^) T cells, effector memory (CD28^–^CD95^+^) T cells, and naive (CD28^+^CD95^–^) T cells. Significance is shown in proportion among species. (**E**) Projections of central memory, effector memory, and naive CD8^+^ T cells onto UMAP dimensional reduction, with division by metaclustering as defined in **B** from 4 different species. (**F**) Proportion of CD4^+^ T cells made comprised of central memory T cells, effector memory T cells, and naive T cells. Significance is shown in proportion among species. (**G**) Projections of central memory, effector memory, and naive CD4^+^ T cells onto UMAP dimensional reduction, with division by metaclustering as defined in **B** from 4 different species. (**H**) Proportion of CD8^+^ T cells that are central memory plotted against human-equivalent age. (**I**) Proportion of CD8^+^ T cells that are effector memory plotted against human-equivalent age. (**J**) Proportion of CD4^+^ T cells that are naive plotted against human-equivalent age. The asterisks indicate significant differences among species as calculated by 2-way ANOVA with Šidák’s multiple comparison analysis. *P < 0.05; **P < 0.01; ***P < 0.001; ****P < 0.0001. The number of green monkeys (GM), crab-eating macaques (CEM), humans, and rhesus monkeys (RhM) are 2, 3, 2, and 3, respectively, for **A**, **C**, and **E**–**G** and are 4, 3, 4, and 3, respectively, for **D**, **F**, and **H**–**J**. NHP, nonhuman primate; UMAP, Uniform Manifold Approximation and Projection.

The Uniform Manifold Approximation and Projection (UMAP) dimensional reduction revealed varying degrees of overlap among cell clusters in PBMCs across different species (Figure 1A). T cell populations, including CD4^+^ and CD8^+^ T cells, exhibited a high degree of similarity between human and NHP samples. However, a notable distinction was observed between the populations of B cells and myeloid cells in all samples. Human cells were identified as the predominant component in specific metaclusters (MC-2: IgD^+^ B cells; MC-16: CD11c^+^ DCs) through metaclustering analysis utilizing FlowSOM (Figure 1B and Supplemental Figure 4). The CD4^+^ T cell metacluster MC-13 and the myeloid cell metaclusters MC-7 and MC-10 exhibited distinct separation between human and NHPs via UMAP visualization yet were aggregated together by FlowSOM analysis. The metaclusters in the 3 different NHP species showed an abundance of other cluster types. GMs had an increased MC-3 component relative to the rest of the metaclusters (Figure 1C). Additionally, marker intensity projected onto the UMAP showed differences in CD11c, CD38, CD45, and IgD among clusters that were predominantly human and those that were predominantly NHPs (Supplemental Figure 5).

### Dynamics of memory cell population among species in PBMCs.

Naive and memory subsets of CD4^+^ and CD8^+^ T cells were identified using bivariate traditional gating (Supplemental Figure 2). Different cell subsets including T cells, B cells, DCs, myeloid cells, and monocytes can also be defined by traditional gating strategy in PBMCs (Supplemental Figure 6). To increase statistical power, additional human and GM PMBC samples were analyzed in subsequent runs together with the initial samples. Due to batch effects, these samples were not included in the aforementioned unbiased analysis. Significant differences were identified in the naive and memory proportions of CD4^+^ and CD8^+^ T cells among species (Figure 1, D–G). CEMs had a significantly smaller percentage of circulating CD8 central memory T cells than RhMs (5.4% versus 32.8%, *P* = 0.0132) and a higher percentage of circulating CD8^+^ effector memory T cells (91.7%) than humans (26.6%, *P* < 0.0001), GMs (63.2%, *P* = 0.0096), or RhMs (63.3%, *P* = 0.017). Humans had a higher percentage of naive CD8^+^ T cells (38.25%) than GMs (1.1%, *P* = 0.0002), RhMs (8.1%, *P* = 0.0057), or CEMs (0.7%, *P* = 0.0005) (Figure 1, D and E). The locations of each CD8^+^ T cell subtype were projected on the UMAP figure with metaclustering (Figure 1E). CEMs were observed to have a higher proportion of circulating CD4 effector memory T cells than humans (46.4% versus 1.4%, *P* = 0.0075) or GMs (46.4% versus 0%, *P* = 0.0056; Figure 1F). CEMs were noted to have a significantly lower percentage of naive CD4^+^ T cells than humans (1.7% versus 45.6%, *P* = 0.0093) or RhMs (1.7% versus 40.7%, *P* = 0.0381; Figure 1F). CD4^+^ T cell subtypes were additionally projected onto the UMAP figure utilizing metaclustering, analogous to the CD8^+^ T cell population (Figure 1G).

### Role of aging in T cell subtype proportionality.

Given that the relative proportions of T cell subtypes are known to undergo changes with aging, we subsequently investigated whether this phenomenon was evident in our dataset. Utilizing published age equivalencies for NHP (17, 18), we plotted adjusted age against the proportion of each T cell subtype and determined significant effects through Pearson correlation analysis. A significant correlation was observed between the percentage of naive CD4^+^ T cells and age (*r* = –0.657, *P* = 0.0203). Trends were observed in correlation across CD8^+^ effector memory and central memory subtypes and age (*r* = 0.557, *P* = 0.0598; *r* = –0.517, *P* = 0.0855, respectively; Figure 1, H–J). No significant correlation with age was observed for other T cell subtypes.

### Performance of the panel across multiple tissue types.

**Figure 2:**
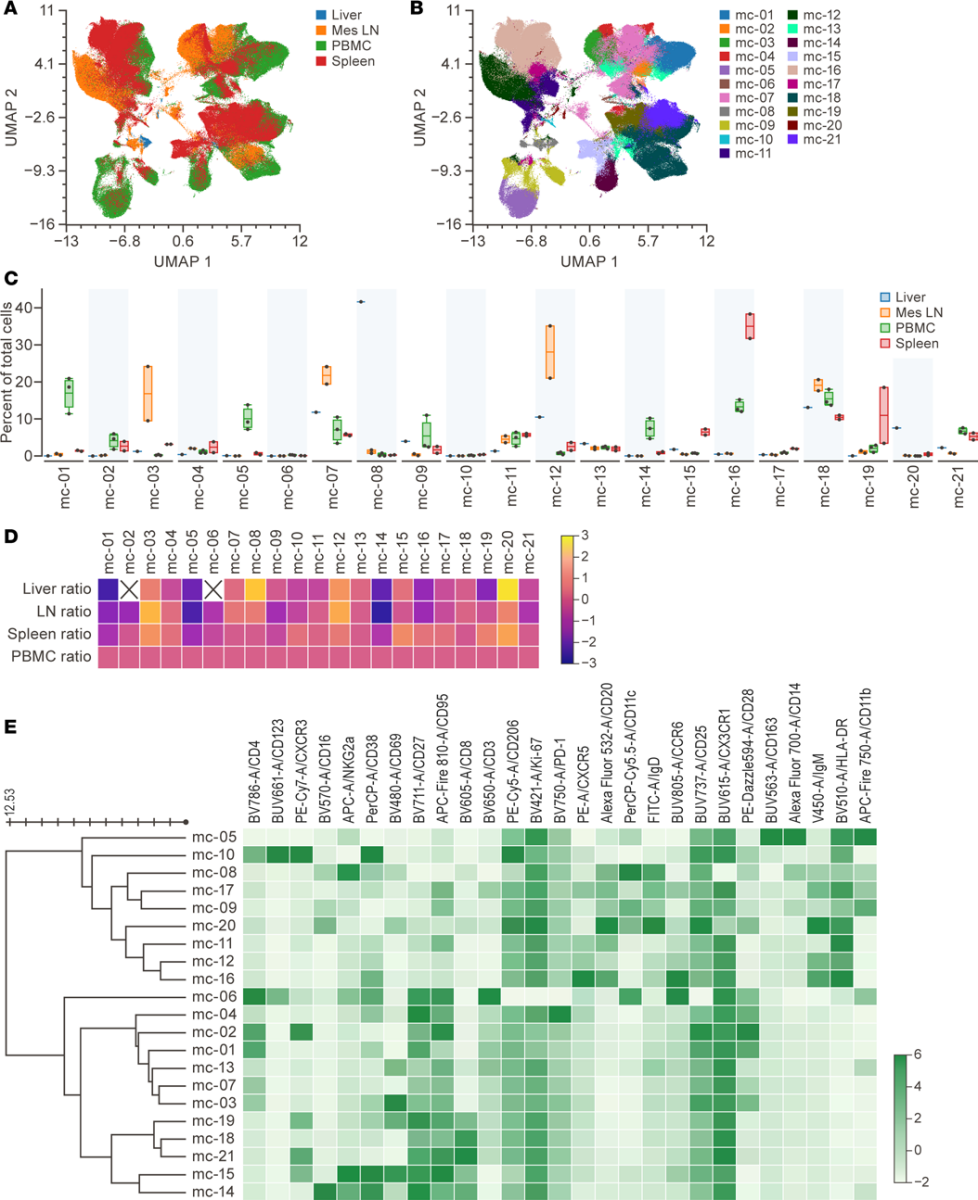
Comparisons of leukocytes from multiple tissues in RhMs. (**A**) UMAP dimensional reduction was performed on live CD45^+^ cells from liver, Mes LN, PBMCs, and spleen of RhMs, revealing overlap of major populations. (**B**) The UMAP plot from **A** shows the FlowSOM metaclusters (*k* = 21) projected onto it. (**C**) Proportions of total cells in each tissue belonging to each metacluster are displayed using column graphs with means. (**D**) Fold change of the proportion of cells in a tissue compared with that in PBMCs for 21 metaclusters plotted on a logarithmic scale. The color scale indicates fold change values, with yellow representing a 2-fold increase and blue representing a 2-fold decrease. (**E**) FlowSOM analysis produced a heatmap with 21 metaclusters. Each row represents a unique metacluster, and columns represent analyzed markers. Mean fluorescence intensity values for metaclusters are represented by color scaling for each marker independently. Mes LN, mesenteric lymph node; RhMs, rhesus monkeys; UMAP, Uniform Manifold Approximation and Projection.

To evaluate the panel performance in cells from multiple tissues, comparisons were made among PBMCs (*n* = 3) and single-cell suspensions made from spleen (*n* = 2), Mes LN (*n* = 2), and liver (*n* = 1) samples obtained at necropsy from RhMs. UMAP dimensional reduction revealed overlap among PBMCs, Mes LN, and spleen samples (Figure 2A). Assessment with FlowSOM identified 21 metaclusters with notable differences in their relative abundance across tissues (Figure 2, B–E).

### Cell phenotypic changes following hemorrhagic fever infection.

**Figure 3:**
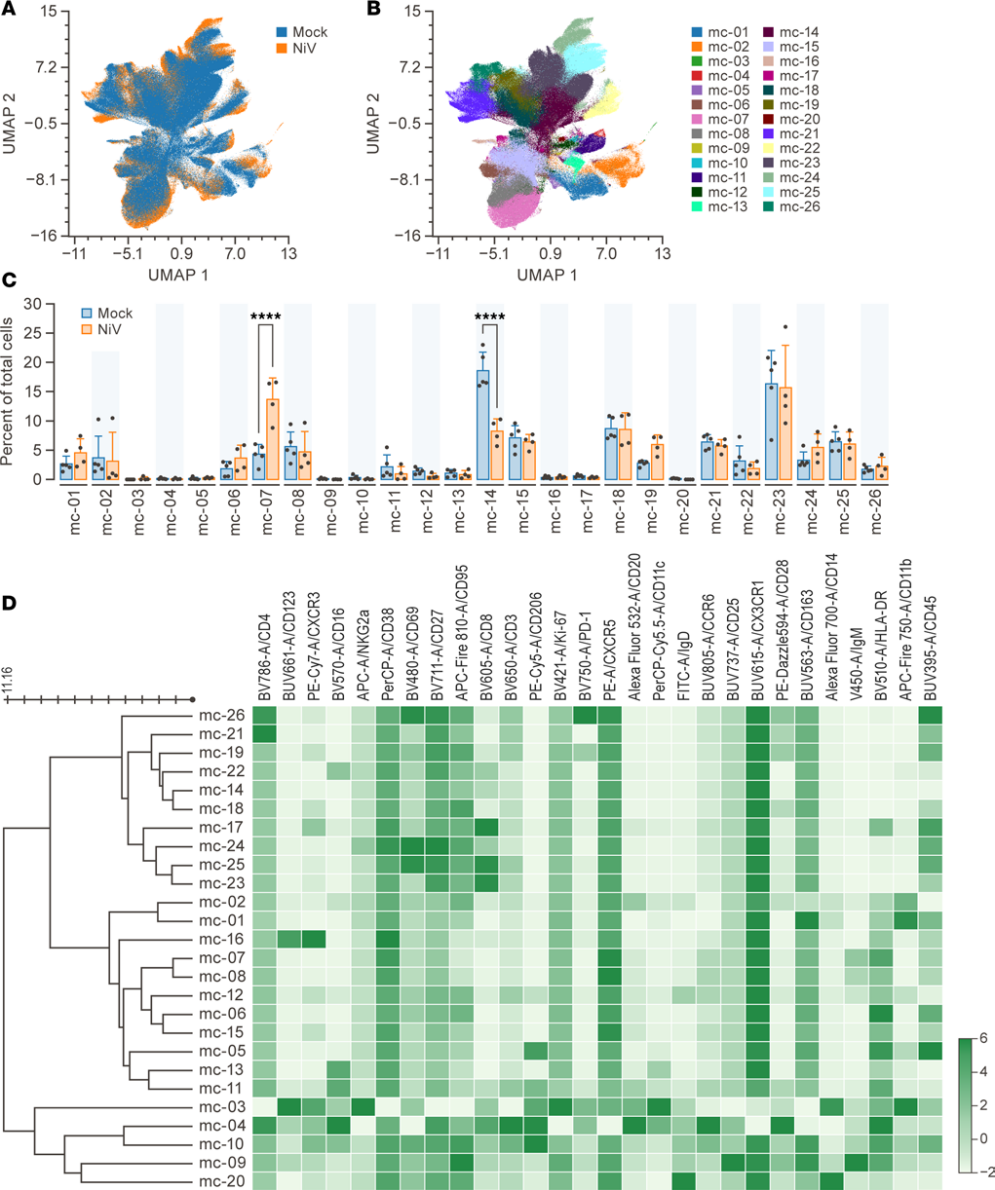
Changes in splenocytes phenotype between exposed and unexposed GMs. (**A**) UMAP dimension reduction of live, CD45^+^ splenocytes from NiV-exposed GMs (*n* =4) versus splenocytes from unexposed GMs (*n* = 5). (**B**) The UMAP plot from **A** shows the FlowSOM metaclusters (*k* = 26) projected onto it. (**C**) Proportions of total cells from each group belonging to each metacluster are displayed using column graphs with means. Note the following metacluster for specific cell types: MC-7, IgM^+^ B cells, MC-14, CD28^–^CD95^+^, CD8^+^ effector memory T cells. (**D**) FlowSOM analysis produced a heatmap with 26 metaclusters. Each row represents a unique metacluster, and columns represent analyzed markers. Mean fluorescence intensity values for metaclusters are represented by color scaling for each marker independently. The asterisks indicate significant differences among species as calculated by 2-way ANOVA with Šidák’s multiple comparison analysis. ****P < 0.0001. GMs, green monkeys; NiV, Nipah virus; UMAP, Uniform Manifold Approximation and Projection.

To evaluate the panel’s efficacy in analyzing specimens from viral hemorrhagic diseases, such as NiV, a comparative analysis was conducted between splenocytes derived from NIV-exposed GMs (*n* = 4) and splenocytes obtained from unexposed GMs (*n* = 5). After using UMAP dimensional reduction and FlowSOM metaclustering, we identified several metaclusters that had different proportions between infected and uninfected splenocytes (Figure 3, A–C). Metaclusters were defined using relative expression of all markers (Figure 3D). MC-7 IgM^+^ B cells were observed to increase in proportion after NiV infection as compared with control splenocytes (13.7% versus 4.36%, *P* < 0.0001), while MC-14, identified as CD8^+^ effector memory T cells (CD28^–^CD95^+^) were observed to decrease in proportion as compared with controls (8.26% versus 18.6%, *P* < 0.0001).

**Figure 4:**
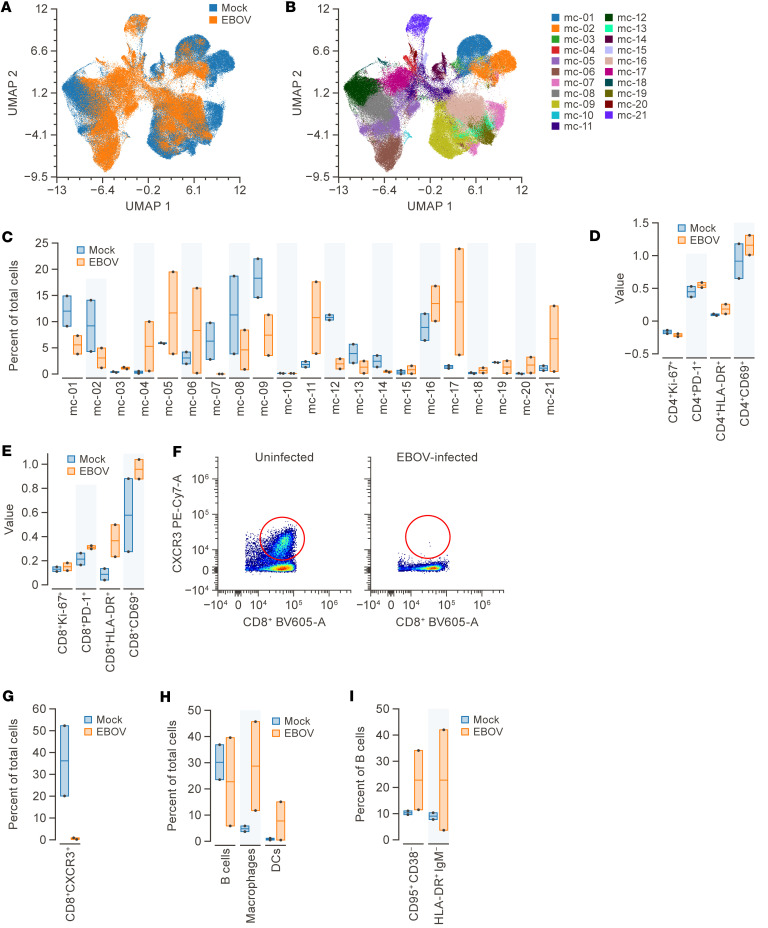
Comparison of Mes LN cell phenotypes in RhMs with and without EBOV exposure. (**A**) UMAP dimension reduction of live CD45^+^ cells from Mes LN of EBOV-exposed versus unexposed animals (*n* =2). (**B**) The UMAP plot from **A** shows the FlowSOM metaclusters (*k* = 21) projected onto it. (**C**) Proportion of total cells from each group belonging to each metacluster are displayed using column graphs with means. (**D**) Expression of proliferation marker (Ki67), T cell exhaustion marker (PD-1), early activation marker (CD69), and late activation marker (HLA-DR) in Mes LN CD4^+^ T cells from EBOV-exposed versus unexposed RhMs. (**E**) Expression of Ki-67, PD-1, HLA-DR, and CD69 on CD8^+^ T cells in EBOV-exposed versus unexposed RhMs. (**F**) Representative dot plots of CD8^+^ T cells expressing CXCR3^+^ cells are shown for unexposed (left panel) and EBOV-exposed (right panel) samples. Circled areas represent CXCR3^+^CD8^+^ T cells. (**G**) The frequency of CXCR3^+^CD8^+^ T cells is presented for EBOV-exposed and unexposed RhMs in columns with means. (**H**) The proportion of CD20^+^ B cells, CD11b^+^ macrophages, and CD11b^+^CD11c^+^ DCs in Mes LN from EBOV-exposed versus unexposed RhMs. (**I**) The proportion of CD20^+^ B cells that are CD95^+^, CD38^–^, HLA-DR^+^, and IgM^–^ in Mes LN from EBOV-exposed versus unexposed RhMs in columns with means (*n* =2). EBOV, Ebola virus; Mes LN, mesenteric lymph node; RhMs, rhesus monkeys; UMAP, Uniform Manifold Approximation and Projection.

An additional comparison was also made between cells isolated from Mes LN of RhM that were either unexposed (*n* = 2) or exposed to EBOV (*n* = 2). The results showed acceptable overlap between the 2 sample types using UMAP dimensional reduction (Figure 4A). FlowSOM metaclustering identified 21 metaclusters with relative proportion changes between the 2 groups (Figure 4, B and C). Expression of proliferation marker (Ki67) (19), T cell exhaustion marker (PD-1) (20), early activation marker (CD69) (21), and late activation marker (HLA-DR) (21) in Mes LN CD4^+^ T cells from EBOV-exposed and unexposed groups were analyzed. However, we found no difference in the expression level of those markers between the 2 groups (Figure 4D). In contrast, RhMs exposed to EBOV had higher expression levels of the markers PD-1, HLA-DR, and CD69 on CD8^+^ T cells compared with unexposed controls (Figure 4E). Further examination of CD8^+^ T cells from the Mes LN revealed that CXCR3^+^ cells were absent in EBOV-exposed RhMs, whereas they represented approximately 35% of CD8^+^ T cells in unexposed animals (Figure 4, F and G). Macrophages and DCs also represented a higher proportion of total cells in the Mes LN of EBOV-exposed animals as compared with unexposed controls (Figure 4H). Heterogeneity was observed in the B cell response to EBOV infection; 1 animal had very few CD20^+^ B cells (5.9%) as a proportion of total cells isolated from the Mes LN (Figure 4H). The remaining animal had higher proportions of CD95^+^CD38^–^ B cells as well as a large proportion of HLA-DR^+^IgM^–^ B cells as compared with B cells from the Mes LN of unexposed controls (Figure 4I).

## Discussion

A flow cytometry panel that can work for various species would serve as an essential tool in evaluating disease pathogenesis and assessing medical countermeasures. This panel can identify the frequency and fluorescence intensity of immune cells across species without the need to change fluorescent colors and clones, thus avoiding variations caused by different fluorochromes and clones. Currently, there is not enough documentation about usage of the same multicolor panel for analysis of NHP and human samples; such a panel could provide a foundational link for preclinical and clinical data. In this study, we developed a 29-color panel that can be used across species, including human clinical samples, by changing only 2 monoclonal antibodies while maintaining the ability to define major immune cell populations. Our analysis shows that it is possible to create an inclusive flow cytometry panel with minimal spectral overlap, despite challenges associated with the lack of unique fluorophores and cross-reactive antibodies. The highest SIM was 0.85, lower than detected from 40- and 43-color panels (15, 16). For accurate unmixing, a SIM value of 0.98 or less is suggested using single-stained reference controls (15).

This 29-color flow cytometry panel allows for a detailed examination of T cells, including their CD4 and CD8 markers, as well as their various subtypes (such as naive, central memory, effector memory, proliferation, exhaustion, early activation, and late activation markers). The panel also enables the identification of different subsets of B cells (including naive, switched memory, nonswitched memory, IgM^+^ memory cells, and plasmablasts) along with NK cells, DCs (both plasmacytoid and monocyte-derived), macrophages, monocytes (classical, intermediate, and nonclassical), and cells that express chemokine receptors (specifically, CX3CR1, CCR6, CXCR3, and CXCR5). We observed differences in the expression of memory cell markers between NHP and human samples by using the traditional gating strategy. The disparity in abundance between naive and memory T cell subsets among NHPs may be attributed to the differential expression of these markers across species. Comprehensive functional studies or RNA-Seq analyses of the sorted naive and memory cell populations could elucidate the underlying mechanisms for the CD28 and CD95 expression differences that are utilized to define naive and memory T cells. This aspect warrants further investigation. The UMAP analysis identified similarities and differences in phenotypic marker expression across species and tissues in flow cytometry data. The analysis generated several metaclusters that can be further examined in different stages of disease. A significant negative correlation was observed between CD4^+^ naive T cells and age, while inverse and positive correlations were found between age and central memory CD8^+^ T cells and between age and effector memory CD8^+^ T cells. These findings suggest that, as age increases, the population of naive CD4^+^ T cells and central memory CD8^+^ T cells decreases, leading to the expansion of effector memory CD8^+^ T cells, a phenomenon that has been corroborated by previous studies (22–25). The presence of additional metaclusters in human PBMCs compared with those of NHPs indicates that NHP models may not serve as a comprehensive animal model for understanding human disease pathogenesis. This observation aligns with a previous study that demonstrated significant differences in whole blood cell phenotypes between humans and NHPs (26). Given the presence of distinct metaclusters among species, it is imperative not to underestimate or prioritize one model over another but rather to consider the limitations of the animal model or cell population that may significantly affect the understanding of disease pathogenesis or the development of vaccines and drugs.

Viral infection induces a humoral immune response by stimulating B cell proliferation, thereby generating antibodies, including neutralizing antibodies. A significant elevation of IgM^+^ B cells in NiV-exposed GMs at 20–57 days after exposure in splenocytes suggests that the survival of these animals may be associated with the upregulation of IgM^+^ B cells. However, the presence of memory B cells, as well as neutralizing and nonneutralizing antibodies, has not been monitored in these animals, which could provide evidence of their survival. Further information lies in human clinical examples; elevated B cell counts, along with the generation of NiV-specific IgG and IgM antibodies, were identified as contributing factors to the recovery of one NiV infection survivor in India (27). The presence of NiV-specific memory B cell responses in a subset of NiV infection survivors 25 years after the outbreak in Malaysia suggests that anti-NiV humoral immune responses may be a critical factor for recovery (28). The study did not include measurements of circulating IgM^+^ B cells, focusing exclusively on tissue-specific observations. In NiV-exposed animals, the marked reduction in splenic CD8 effector memory T cells indicates that, even after recovery, tissue-specific effector memory CD8^+^ T cells could not be reconstituted. Conversely, prior observations in a GM 36 days after exposure demonstrated an increase in peripheral CD8^+^ effector memory T cells relative to the baseline time point (29). This discrepancy suggests that the dynamics of effector memory CD8^+^ T cells may differ between tissues and circulation, warranting further investigation.

Increased PD-1 expression in EBOV-exposed RhMs suggests that exhausted CD8^+^ T cells may be incapable of inducing protective cytolytic function. Significant PD-1 expression enhancement was associated with EBOV reactivation and was found in clinical samples from cases with fatal outcomes (30, 31). In EBOV infection, activation markers were observed to have increased early (CD69) and late (HLA-DR), consistent with previous findings in EBOV-exposed RhMs (24) and in patients with EVD (22). These findings suggest the presence of virus-mediated CD8^+^ T cell activation after infection. CXCR3 is highly expressed in effector T cells and plays a crucial role in regulating trafficking and function of T cells (32). In a previous study on EBOV-exposed RhMs, it was suggested that the decrease in circulating CXCR3^+^ T and B cells may be attributed to the migration of these cells into inflamed lymphoid tissues (33). However, our study on EBOV-exposed RhMs showed that the reduction of CXCR3^+^ T cells in the Mes LN contradicts the earlier hypothesis that trafficking to lymph nodes was taking place. Unfortunately, T cells from organ tissue samples were not sufficient to determine if CXCR3^+^ T cells were present outside lymphoid tissue.

This study was limited by the small sample size of unexposed and exposed animals in each group. While our investigation provides insights into lymphoid tissues in NHP models of fatal EVD and late-stage NiV infection, it does not elucidate the infection dynamics during the early or late acute phases of disease. A recent study demonstrated alterations in circulating cell populations, enhanced cell proliferation, increased expression of IFN-α, IFN-γ, and Myxovirus resistance 1 (MX1), and reduced expression of MHC class II genes in monocytes through single-cell transcriptomics and CyTOF-based single-cell protein expression analysis in an EBOV-exposed RhM model (34). Investigations into the phenotypic characterization and functional responses of circulatory and lymphoid tissues at various stages of viral infection will provide a comprehensive understanding of the phenotypic differentiation of major cell populations and complement our knowledge of the significance of different subsets in disease conditions. A further limitation of this study is the absence of human samples from patients with NiV infection or EVD at different stages of disease for comparison with control groups. Despite these limitations, we have demonstrated the feasibility of using this panel on different species and have shown the robustness of this panel in defining different cell populations using both traditional and nontraditional flow analysis in healthy and disease conditions.

In conclusion, this study presents a comprehensive characterization of a 29-color flow cytometry panel capable of identifying multiple cell types (T cells, B cells, monocytes, macrophages, DCs) and their subsets and phenotypes utilizing both human and NHP samples. The panel comprises 27 markers that demonstrated a high degree of cross-reactivity with PBMCs and tissues from 3 different NHPs as well as patients. In human samples, 2 antibodies, CD45 and CD19/CD20, were employed as replacement antibodies while maintaining the same fluorophore. This panel serves as a crucial tool for defining and characterizing diverse cell populations in both humans and NHPs, without necessitating alterations to the panel, thereby facilitating the comparison of data generated from different species in health and disease states.

## Methods

### Sex as a biological variable.

This study included samples from both male and female human participants and animals.

### Study samples.

This study involved PBMCs or tissue-isolated cells from 4 healthy human volunteers, 11 RhMs, 4 CEMs, and 13 GMs (Table 2). The study assessed frozen splenocytes from 4 GMs exposed to the NiV. Each GM was exposed with an achieved dose range of 61.2–773.7 PFU of NiV-Malaysia (accession no. KY425646.1) via aerosol route. The study also analyzed frozen Mes LN cells from 2 RhMs exposed to EBOV (Table 2). The animals were inoculated with an achieved dose of 1,300 PFU of EBOV/H sapiens-tc/GIN/2014/Makona-C05 (accession no. KX000400) intramuscularly.

### Isolation of PBMCs.

PBMCs were isolated from EDTA-anticoagulated whole blood using AccuSpin tubes (MilliporeSigma) with Histopaque-1077 (MilliporeSigma) as a density gradient media. In brief, the diluted whole blood was added to the AccuSpin tubes and spun for 1,000*g* for 10 minutes with the brake off. The layer of PBMCs was then carefully removed, washed, and resuspended in complete RPMI medium with 10% fetal calf serum (cRPMI). The viability and cell count were determined using trypan blue dye and a Nexcelom Cell Counter (Nexcelom Bioscience LLC). Both fresh and frozen PBMCs were used for the study. The PBMCs were frozen in Bambanker serum-free cell freezing medium (Fujifilm Wako Chemicals USA Corporation) and stored in a liquid nitrogen freezer.

### Isolation of splenocytes, Mes LN, and liver cells.

Mes LN tissue samples were collected in cold cRPMI and cut into pieces. A GentleMACS Octo dissociator (Miltenyi Biotec) was used to prepare a single-cell suspension of splenocytes without any enzyme digestion. Next, samples were centrifuged at 550*g* for 5 minutes and filtered through a 100 μm cell strainer. After straining, each sample was washed with PBS with 2 mM EDTA (PBS-2). Splenocytes were then lysed using ACK Lyse buffer (Thermo Fisher Scientific) and washed with PBS-2. Cells from Mes LN were isolated by gently pressing through a 100 μm cell strainer and washing with PBS-2. Splenocytes and Mes LN cells were counted using a Nexcelom cell counter and then frozen for future use.

Liver tissue samples were cut into small pieces and placed into GentleMACS C-Tubes containing PBS-2 media with a final concentration of 1.96 μg/mL of collagenase V (MilliporeSigma) and 98 μg/mL of DNase I (MilliporeSigma). After dissociation, cells were washed with PBS-2 and centrifuged at 550*g* for 5 minutes. Then, they were filtered through 100 μm cell strainers, gently washed with PBS-2, and centrifuged at 60*g* for 3 minutes to remove any debris. After centrifugation, cells were lysed using ACK Lyse buffer, washed, and reconstituted in cRPMI. Finally, isolated cells were counted and then frozen for future use.

### Cell thawing.

Frozen cells were thawed using CryoThaw Tube Adapters (Medax International Inc.) by placing on top of 15 mL conical tubes with 9 mL of cRPMI and diluted benzonase (≥ 50 units per mL) as described previously (33). After centrifugation at 350*g* for 5 minutes, each sample was washed and then resuspended in PBS-2 at 5 × 10^6^ cells per mL.

### Panel design.

Our aim was to create a new flow cytometry panel that would consolidate 4 previously existing panels, providing a more comprehensive and accurate analysis of different cell types found in tissues and across different species while concurrently reducing effort and cost. The new panel can distinguish among T cells, B cells, monocytes, DCs, and tissue-specific macrophages. A concerted effort was placed on the phenotype of monocytes, macrophages, and DCs due to their known involvements in viral life cycles and disease course of numerous BSL-4 pathogens (34–36). We analyzed this panel’s compatibility with cells from peripheral blood and tissue samples from humans, RhMs, and CEMs, and from tissues of NiV-exposed GMs and EBOV-exposed RhMs.

### Titrations and reference controls preparation.

A volume of 50 μL of cell suspension was aliquoted and stained with different concentrations of single-color antibodies (Table 1). For surface staining, the samples were incubated with the antibodies at 4°C for 30 minutes and washed. The cells were resuspended in 150 μL of PBS to prepare for acquisition. For intracellular staining, samples were fixed and permeabilized using BD CytoFix/CytoPerm buffer (BD Biosciences) as previously performed (37). Then, samples were stained with different concentrations of single-color antibodies and incubated at 4°C for 30 minutes. Finally, the cells were washed and resuspended in 150 μL of PBS for data acquisition.

Single-stain reference controls were prepared using UltraComp beads (Thermo Fisher Scientific). For live/dead stain, ARC beads (Invitrogen) were used. For each color, a single-stain reference control was acquired, and an unstained sample was used as a reference control. Controls were acquired once, and the reagents used for experimental samples carried the same lot number as the reference controls.

### Polychromatic cell staining and data acquisition.

Cell staining was performed using standard methods as mentioned earlier (8). To block Fc receptor binding, cells were incubated with Human TruStain FcX (BioLegend) at 4°C for 10 minutes. Then, a surface stain master mix containing all of the surface antibodies was added to each tube and incubated at 4°C for 30 minutes (Table 2). Then, the cells were washed with PBS-2. Cells were fixed using BD CytoFix/CytoPerm and washed with PermWash buffer. For intracellular staining, anti-human Ki67 antibodies were added to the cells and incubated at 4°C for 30 minutes. After incubation, the cells were washed with 1× PermWash buffer, reconstituted in 150 μL of PBS, and prepared for data acquisition. Data acquisition was performed within 1 hour of sample staining using a Cytek Aurora flow cytometer (Cytek Biosciences) equipped with 5 lasers, including ultraviolet, violet, blue, yellow-green, and red lasers. Data from each sample, including single-stained, unstained, and complete 29-color flow, were acquired using SpectroFlo Software. Spectral unmixing identified the fluorescence signal for each fluorophore used in the experiment.

### Flow cytometry data analysis.

Unmixed FCS (embedded format type) files were imported into FlowJo v10 (BD Biosciences). Gating was performed on time, forward and side scatter to identify singlet cells. Leukocytes were identified by forward and side scatter, and then files of live CD45^+^ cells were exported. New FCS files of live CD45^+^ cells were imported into OMIQ (Dotmatics) with associated metadata for species, tissue source, and infection status. Data were scaled and subsampled to 50,000 events per sample. Dimensional reduction was performed with the UMAP algorithm (38) and default settings with all fluorescence signals except CD45-BUV395 and Live/Dead Blue. Metaclustering was performed in 10 × 10 clusters with the FlowSOM algorithm (39) by using all fluorescence signals except those previously mentioned. The elbow method for the within-cluster sum of squares was used to identify *k* for each metaclustering run.

### Statistics.

Statistically significant differences among groups were determined using 2-way ANOVA with Šidák’s multiple-comparison analysis. *P* < 0.05 was considered significant. GraphPad Prism (v10.1.1., GraphPad Software) was used for all statistical analyses and graph generation. Cell phenotypic expression was reported by species or metacluster for various markers using mean and range. A 2-tailed Pearson correlation coefficient analysis was conducted to examine the relationship between the frequency of naive and memory T cells and human-equivalent age.

### Study approval.

Healthy volunteers were recruited from June 1, 2023, to July 1, 2023, and enrolled in the NIH study Samples from Human Subjects to Facilitate Basic, Translational, and Clinical Research (protocol no. 17-CC-0148). The NIH IRB approved the protocol, and all participants provided written informed consent.

The study was performed in the BSL-4 laboratory at the NIH National Institute of Allergy and Infectious Diseases Integrated Research Facility at Fort Detrick (IRF-Frederick). All the animals that were utilized in this research project were treated with care and used in a humane manner in accordance with the U.S. Public Health Service Policy on Humane Care and Use of Animals, The Guide for the Care and Use of Laboratory Animals, and the U.S. Government Principles for the Utilization and Care of Vertebrate Animals Used in Testing, Research, and Training policies. In addition, all animal facilities and programs at the IRF-Frederick are accredited by the Association for Assessment and Accreditation of Laboratory Animal Care International.

### Data availability.

All relevant data are included within the manuscript. All raw data are available in the Supporting Data Values file.

### Author contributions.

SMA and BP conceptualized and designed the study. APP, BB, SMA, and BP analyzed the data. BB, RB, AM, DFPR, SMA, and BP performed experiments and collected data. APP and BP wrote the original draft. MRH and SMA reviewed and edited the manuscript. VJM, MRH, and DSC supported the project with samples. All authors have read and approved the content of the paper.

## Supplementary Material

Supplemental data

Supporting data values

## Figures and Tables

**Table 1:**
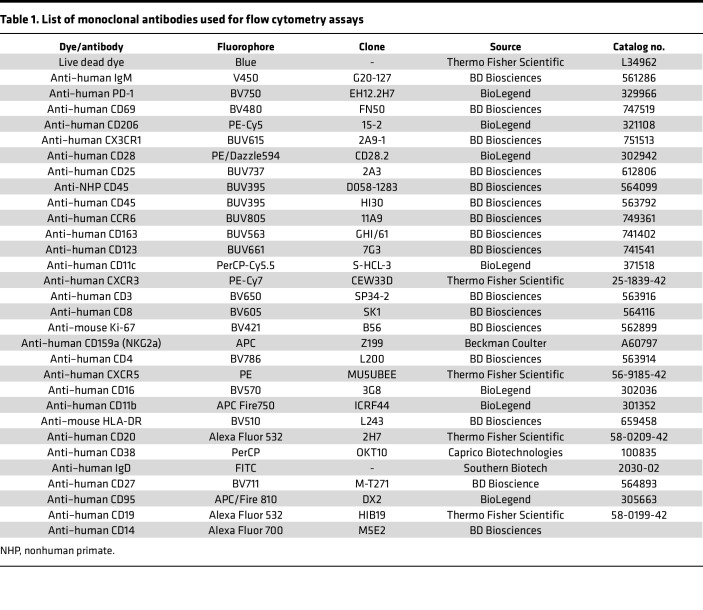
List of monoclonal antibodies used for flow cytometry assays

**Table 2:**
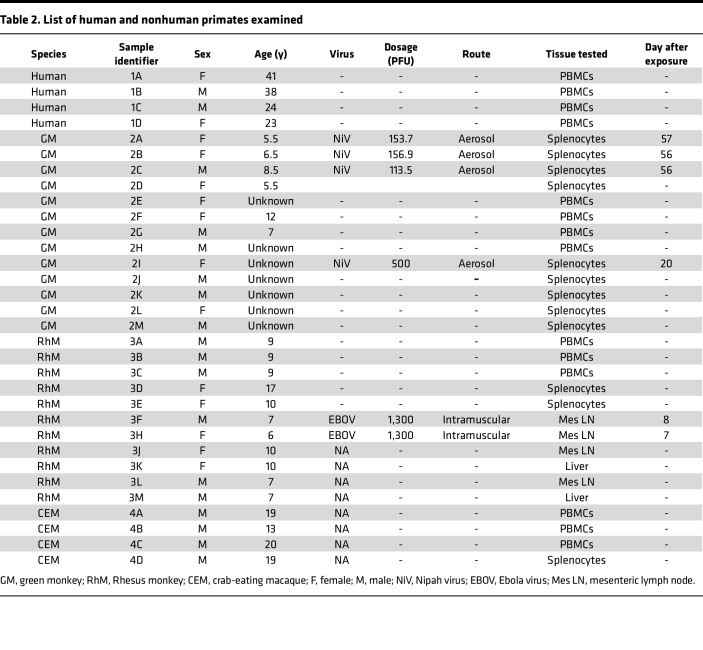
List of human and nonhuman primates examined

## Version 1 04/17/2025

In-Press Preview

## Version 2 06/09/2025

Electronic publication
