# Supplementary material for: Cellular immunophenotyping in human and primate tissues during healthy conditions and Ebola and Nipah infections
Source: JCI Insight. 2025 Apr 17;10(11):e185861. doi: 10.1172/jci.insight.185861 (PMC12220940; doi:10.1172/jci.insight.185861)

Supplemental Figure 1. The complexity index for this 29-color flow cytometry panel is plotted, taking into account the measures of uniqueness (similarity indices) for the entire combination of dyes within the panel. The similarity index among fluorochromes is represented by a color scale with a range of 0 to +1.

Supplemental Figure 2. Gating strategy for detecting naïve and memory T cells from representative rhesus monkey PBMCs. Cells were gated on time, singlets, leukocytes, CD45+ cells, live cells, and CD3+ T cells and subsequently on CD3+CD4+ and CD3+CD8+ T cell subsets. The naïve (CD28+CD95-), central memory (CD28+CD95+) and effector memory (CD28-CD95+) cells for both CD4 and CD8 T-cell subsets are shown in each box of the plots.

Supplemental Figure 3. Histograms for each fluorescent marker, forward scatter, side scatter, and autofluorescence are shown, separated by species, and gated on live and CD45+ PBMCs. Human PBMCs were used as unstained controls. GM, green monkey; RhM, rhesus monkey; CEM, crab-eating macaque.

Supplemental Figure 4: FlowSOM analysis produced a heatmap with 17 metaclusters of total live, CD45+ PBMCs. Each row represents a unique metacluster, and columns represent analyzed markers. Mean fluorescent intensity values for metaclusters are color-scaled for each marker independently; range -2 to 6.

Supplemental Figure 5. Uniform Manifold Approximation and Projection (UMAP) projections overlaid with staining intensity for each fluorescent marker. All plots are of all live CD45+ PBMCs.

Supplemental Figure 6. A traditional gating strategy displays various cellular populations of interest in PBMCs from a rhesus monkey, with the name and frequencies of each population shown in the plots.

Supplemental Figure 1

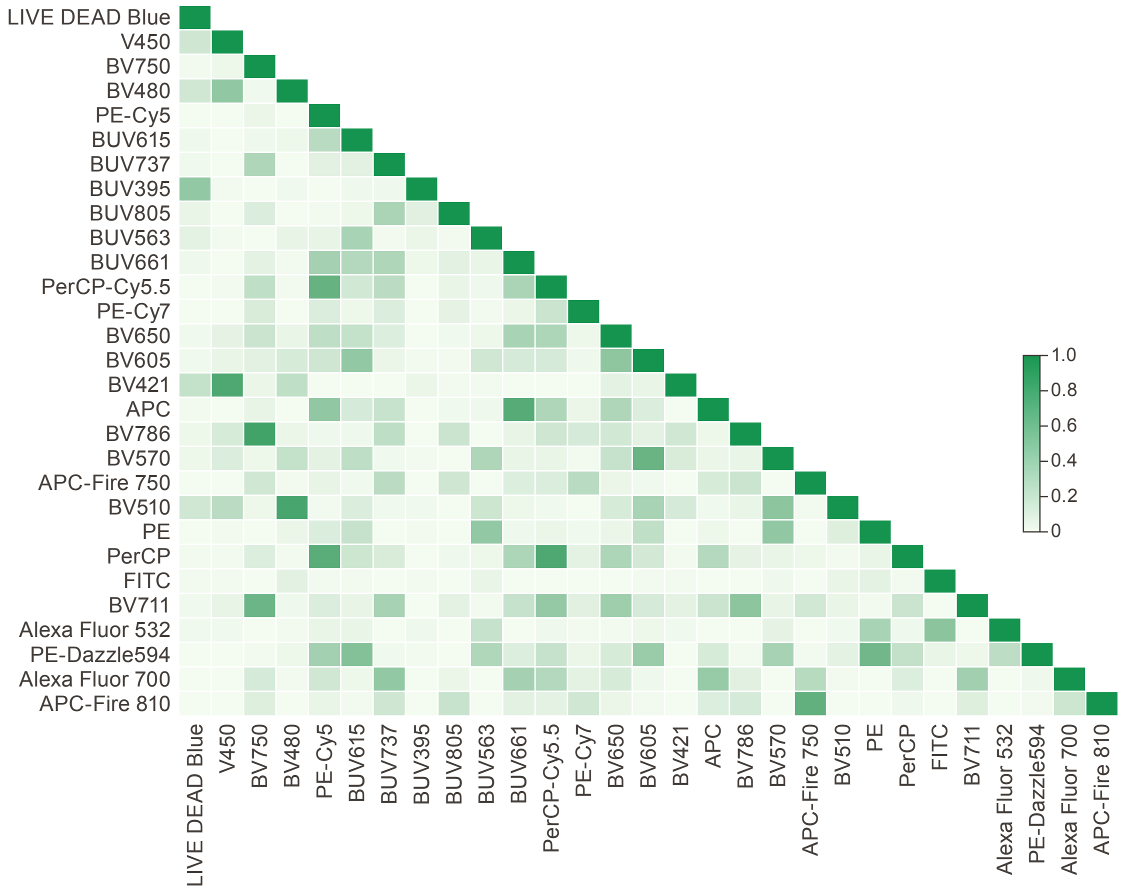

Supplemental Figure 2

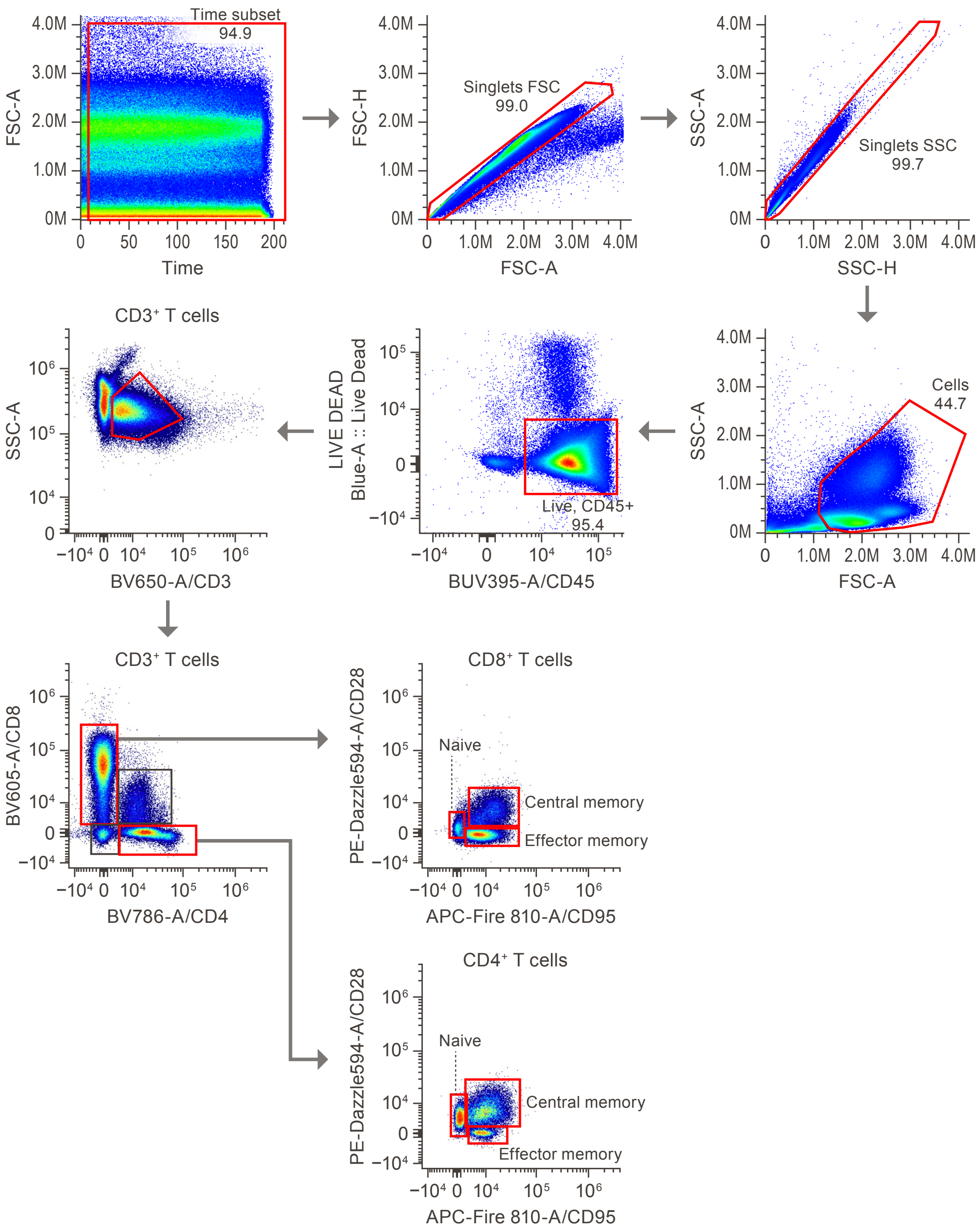

Supplemental Figure 3

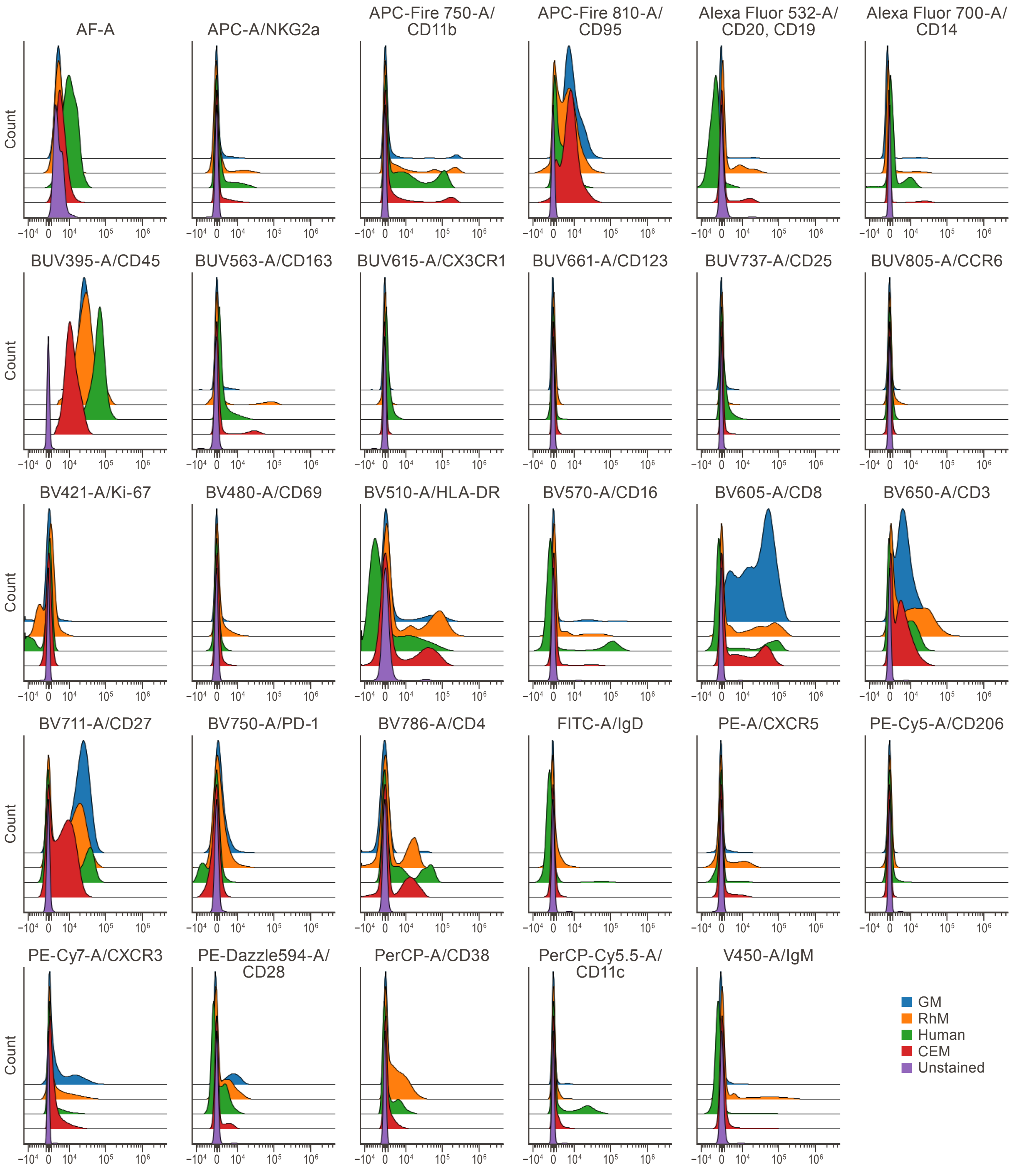

Supplemental Figure 4

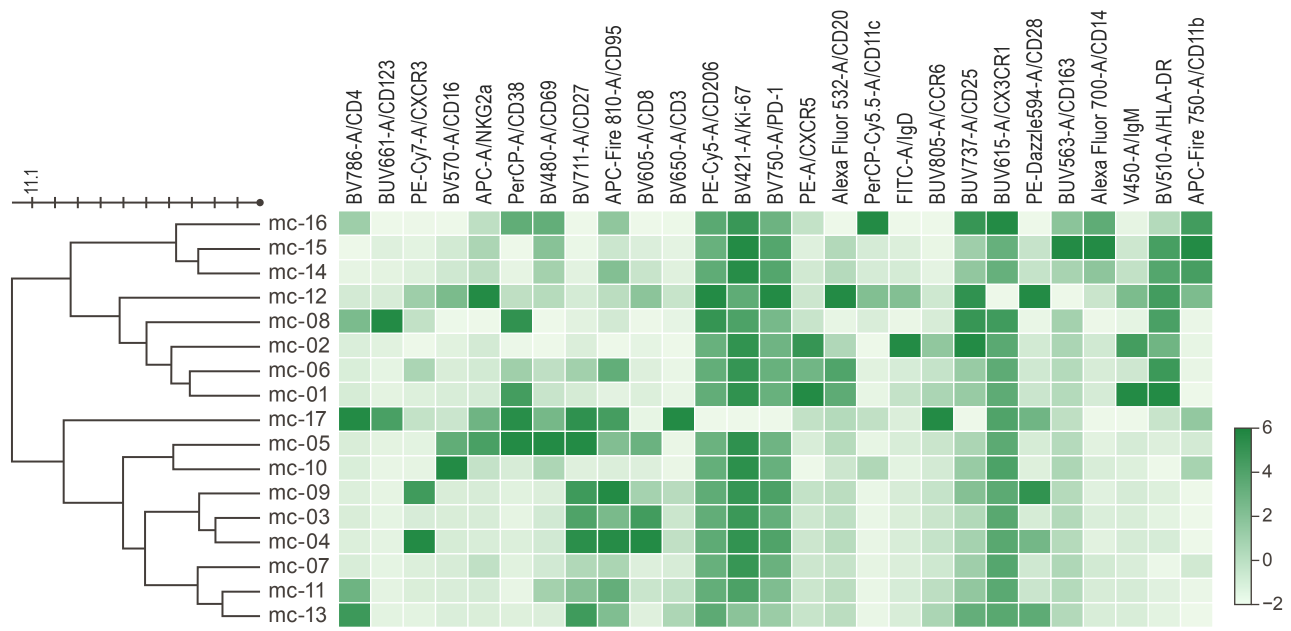

Supplemental Figure 5

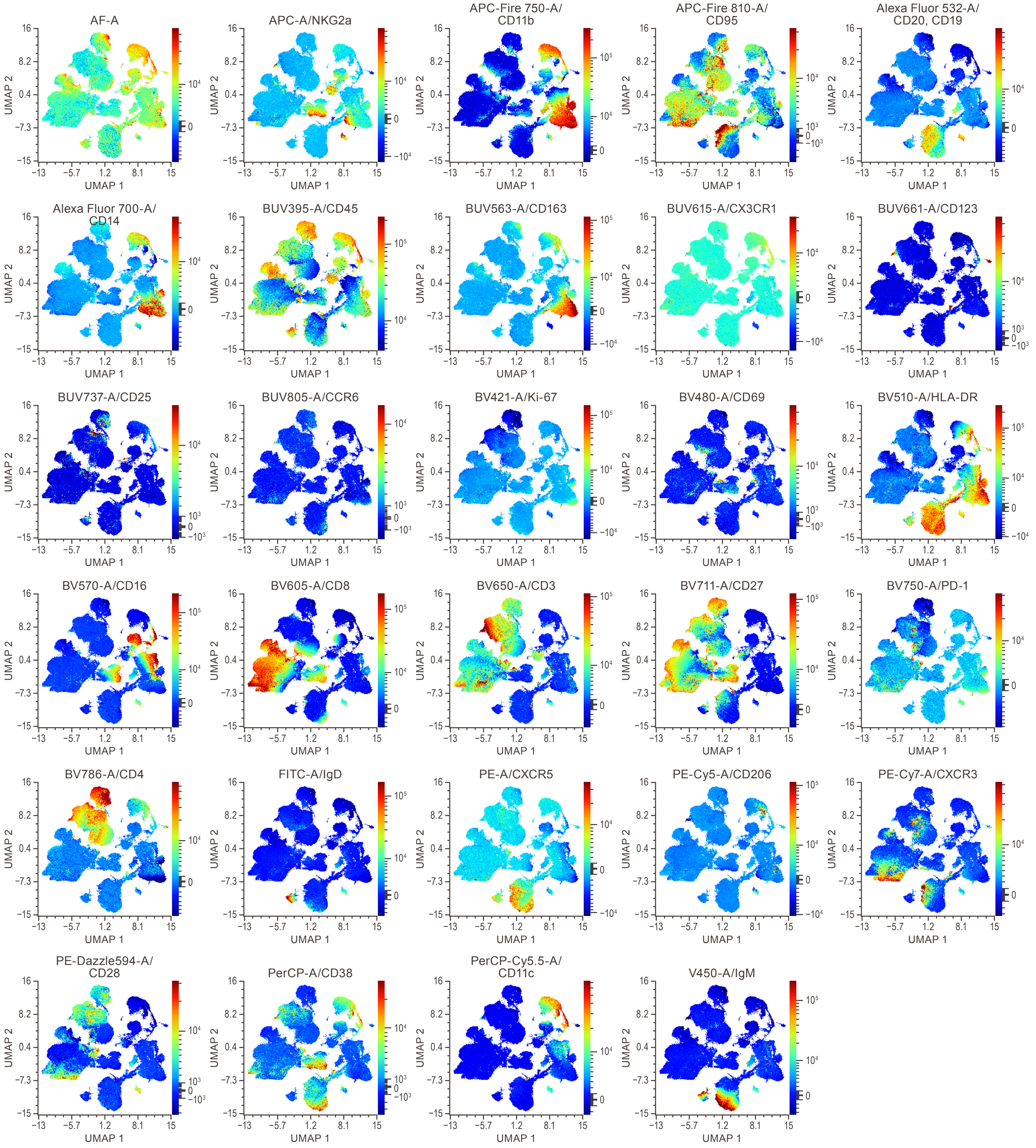

Supplemental Figure 6

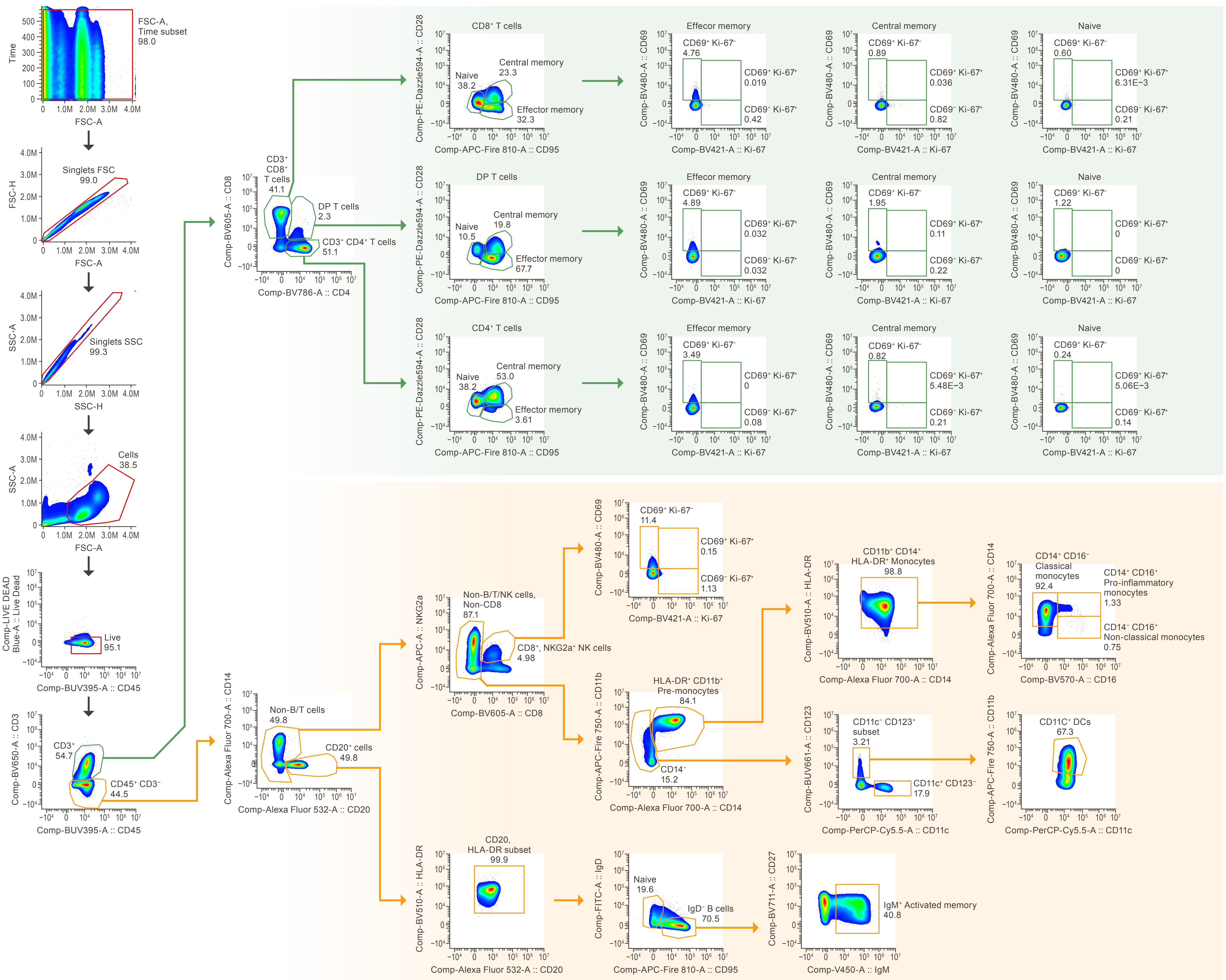

Supplement: Supplemental data [file jciinsight-10-185861-s189.pdf]
